# 4-Bromo­anilinium perchlorate 18-crown-6 clathrate

**DOI:** 10.1107/S1600536810040481

**Published:** 2010-10-20

**Authors:** Min Guo, Min Min Zhao

**Affiliations:** a Ordered Matter Science Research Center, College of Chemistry and Chemical, Engineering, Southeast University, Nanjing 211189, People’s Republic of China

## Abstract

The reaction of 4-bromo­aniline, 18-crown-6, and perchloric acid in methanol yields the title compound, C_6_H_7_BrN^+^·ClO_4_^−^·C_12_H_24_O_6_, in which the protonated –NH_3_^+^ group forms three bifurcated N—H⋯O hydrogen bonds to the O atoms of the crown ether.

## Related literature

For similar crown ether clathrates, see: Akutagawa *et al.* (2002 ▶); Ge *et al.* (2010 ▶); Zhao (2010 ▶). For their ferroelectric properties, see: Zhang, Cheng *et al.* (2009 ▶); Zhang, Ye *et al.* (2009 ▶); Ye *et al.* (2009 ▶). For related structures, see: Ge & Zhao (2010*a* ▶,*b* ▶); Zhao & Qu (2010*a* ▶ *b* ▶). 
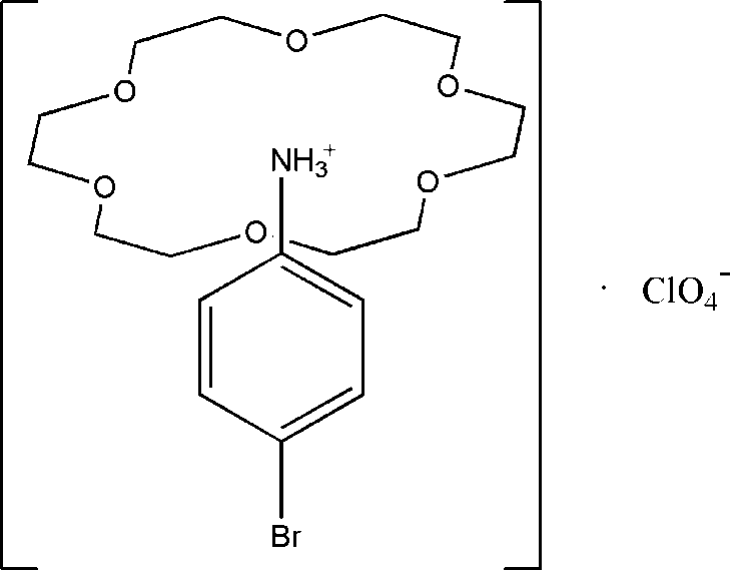


## Experimental

### 

#### Crystal data

C_6_H_7_BrN^+^·ClO_4_^−^·C_12_H_24_O_6_
*M* *_r_* = 536.79
Orthorhombic, 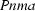

*a* = 15.583 (7) Å
*b* = 11.469 (5) Å
*c* = 12.633 (6) Å
*V* = 2257.7 (18) Å^3^
*Z* = 4
Mo *K*α radiation
μ = 1.99 mm^−1^
*T* = 93 K
0.20 × 0.20 × 0.20 mm

#### Data collection

Rigaku SCXmini diffractometer
Absorption correction: multi-scan (*CrystalClear*; Rigaku, 2005 ▶) *T*_min_ = 0.671, *T*_max_ = 0.678
23692 measured reflections
2694 independent reflections
2561 reflections with *I* > 2σ(*I*)
*R*_int_ = 0.046

#### Refinement

*R*[*F* ^2^ > 2σ(*F* ^2^)] = 0.038
*wR*(*F* ^2^) = 0.104
*S* = 1.01
2694 reflections
146 parameters
H-atom parameters constrained
Δρ_max_ = 0.57 e Å^−3^
Δρ_min_ = −0.50 e Å^−3^

### 

Data collection: *CrystalClear* (Rigaku, 2005 ▶); cell refinement: *CrystalClear*; data reduction: *CrystalClear*; program(s) used to solve structure: *SHELXS97* (Sheldrick, 2008 ▶); program(s) used to refine structure: *SHELXL97* (Sheldrick, 2008 ▶); molecular graphics: *SHELXTL/PC* (Sheldrick, 2008 ▶); software used to prepare material for publication: *SHELXL97*.

## Supplementary Material

Crystal structure: contains datablocks I, global. DOI: 10.1107/S1600536810040481/jh2210sup1.cif

Structure factors: contains datablocks I. DOI: 10.1107/S1600536810040481/jh2210Isup2.hkl

Additional supplementary materials:  crystallographic information; 3D view; checkCIF report

## Figures and Tables

**Table 1:** Hydrogen-bond geometry (Å, °)

*D*—H⋯*A*	*D*—H	H⋯*A*	*D*⋯*A*	*D*—H⋯*A*
N1—H1*A*⋯O2	0.91	2.13	2.864 (2)	137
N1—H1*A*⋯O3	0.91	2.18	2.938 (2)	140
N1—H1*B*⋯O4	0.91	2.10	2.850 (3)	139
N1—H1*B*⋯O3^i^	0.91	2.20	2.938 (2)	138
N1—H1*C*⋯O2^i^	0.91	2.10	2.864 (2)	141
N1—H1*C*⋯O1	0.91	2.18	2.875 (3)	133

Symmetry code: (i) 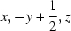.

**Fig. 1.**
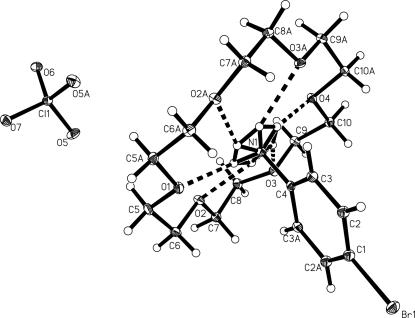
The molecular structure of the title compound, with the atomic numbering scheme. Displacement ellipsoids are drawn at the 30% probability level.

**Fig. 2.**
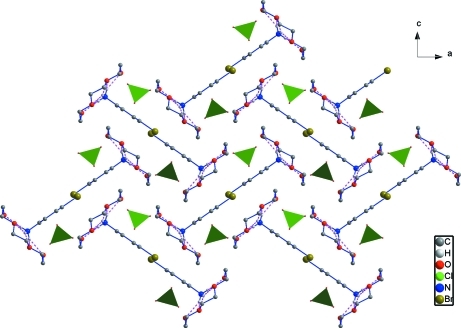
A view of the packing of the title compound, stacking along the b axis. Dashed lines indicate hydrogen bonds.

## supplementary crystallographic information

### Comment

There is currently much interest in crown ethers due to their ability to form non-covalent, H-bonding complexes with ammonium cations both in solid and in solution. Not only the size of the crown ether, but also the nature of the ammonium cation (–NH_4_^+^, RNH_3_^+^, *R*_2_NH_2_^+^, *etc*) can influence on the stoichiometry and stability of these host–guest complexes (Zhao *et al.* 2010). The host molecules combine with the guest species by intermolecular interaction, and if the host molecule possess some specific sites, it is easy to realise high selectivity in ion or molecular recognitions. 18-Crown-6 have the highest affinity for ammonium cation RNH_3_^+^.

Dielectric permittivity of the title compound is tested to systematically investigate the ferroelectric phase transitions materials (Ye *et al.*, 2009; Zhang *et al.*, 2009). The title compound has no dielectric anomaly with the value of 3.5 and 7.8 under 1*M* Hz in the temperature from 80 to 430 K (m.p.> 453 K), suggesting that the compound should be no distinct phase transition occurred within the measured temperature range.

The title compound is composed of cationic [C_6_BrNH_7_(18-Crown-6)]^+^ and one single anionic [ClO_4_]^-^ anions (Fig. 1). Supramolecular rotators was assembled between protonated 4-bromoaniline (C_6_BrH_4_—NH_3_)^+^and 18-crown-6 by of hydrogen-bonding. The ammonium moieties of (–NH_3_^+^) cations were interacted with the oxygen atom of crown ethers through six simple N—H···O hydrogen bonding, forming 1:1 supramolecular rotator-stator structures.

Supramolecular cation structure, [C_6_BrNH_7_(18-Crown-6)]^+^, were introduced as counter cations to [ClO_4_]^-^ anions. The crown adopts a conformation in which the rings show some distortion from the mean plane. The C—N bonds of [C_6_BrNH_7_]^+^ were almost perpendicular to the mean oxygen planes of crown ethers. Cl has a flattened tetrahedral coordination by four O^-^ ions [range of *cis*-bond angles = 109.08 (14)–109.88 (15) °; dav (Cl—O) = 1.444 (2)–1.449 (2) Å].

The title compound was stabilized by intramolecular N—H···O hydrogen bonds, but no intermolecular hydrogen bond was observed (Fig. 2). The intramolecular N—H···O hydrogen bonding length are within the usual range: 2.850 (3) and 2.938 (2) Å.

### Experimental

C_6_BrNH_6_.HClO_4_ (2 mmol, 0.546 g) and 18-crown-6 (2 mmol, 0.528 g) were dissolved in methanol solution. The precipitate was filtered out. Two days later, single crystals suitable for X-ray diffraction analysis were obtained from slow evaporation of methanol solution at 0°C.

### Refinement

All the C—H hydrogen atoms were calculated geometrically and with C—H distances ranging from 0.93 to 0.97 Å and were allowed to ride on the C and O atoms to which they are bonded. With which *U*_iso_(H) = 1.2Ueq(C).

All the N—H hydrogen atoms were calculated geometrically. The positions of the H atoms of the nitrogen atoms were refined using a riding model with N—H = 0.91 Å and *U*_iso_(H) = 1.2*U*_eq_(N).

### Crystal data

**Table:**

C_6_H_7_BrN^+^·ClO_4_^−^·C_12_H_24_O_6_	*F*(000) = 1112
*M**_r_* = 536.79	*D*_x_ = 1.579 Mg m^−^^3^
Orthorhombic, *P**n**m**a*	Mo *K*α radiation, λ = 0.71073 Å
Hall symbol: -P 2ac 2n	Cell parameters from 6002 reflections
*a* = 15.583 (7) Å	θ = 3.1–27.5°
*b* = 11.469 (5) Å	µ = 1.99 mm^−^^1^
*c* = 12.633 (6) Å	*T* = 93 K
*V* = 2257.7 (18) Å^3^	Prism, colorless
*Z* = 4	0.20 × 0.20 × 0.20 mm

### Data collection

**Table:**

Rigaku SCXmini diffractometer	2694 independent reflections
Radiation source: fine-focus sealed tube	2561 reflections with *I* > 2σ(*I*)
graphite	*R*_int_ = 0.046
Detector resolution: 28.5714 pixels mm^-1^	θ_max_ = 27.4°, θ_min_ = 3.1°
CCD_Profile_fitting scans	*h* = −20→20
Absorption correction: multi-scan (*CrystalClear*; Rigaku, 2005)	*k* = −14→14
*T*_min_ = 0.671, *T*_max_ = 0.678	*l* = −16→16
23692 measured reflections	

### Refinement

**Table:**

Refinement on *F*^2^	Primary atom site location: structure-invariant direct methods
Least-squares matrix: full	Secondary atom site location: difference Fourier map
*R*[*F*^2^ > 2σ(*F*^2^)] = 0.038	Hydrogen site location: inferred from neighbouring sites
*wR*(*F*^2^) = 0.104	H-atom parameters constrained
*S* = 1.01	*w* = 1/[σ^2^(*F*_o_^2^) + (0.0645*P*)^2^ + 1.990*P*] where *P* = (*F*_o_^2^ + 2*F*_c_^2^)/3
2694 reflections	(Δ/σ)_max_ = 0.001
146 parameters	Δρ_max_ = 0.57 e Å^−^^3^
0 restraints	Δρ_min_ = −0.49 e Å^−^^3^

### Special details

**Table:**

Geometry. All e.s.d.'s (except the e.s.d. in the dihedral angle between two l.s. planes) are estimated using the full covariance matrix. The cell e.s.d.'s are taken into account individually in the estimation of e.s.d.'s in distances, angles and torsion angles; correlations between e.s.d.'s in cell parameters are only used when they are defined by crystal symmetry. An approximate (isotropic) treatment of cell e.s.d.'s is used for estimating e.s.d.'s involving l.s. planes.
Refinement. Refinement of *F*^2^ against ALL reflections. The weighted *R*-factor *wR* and goodness of fit *S* are based on *F*^2^, conventional *R*-factors *R* are based on *F*, with *F* set to zero for negative *F*^2^. The threshold expression of *F*^2^ > σ(*F*^2^) is used only for calculating *R*-factors(gt) *etc*. and is not relevant to the choice of reflections for refinement. *R*-factors based on *F*^2^ are statistically about twice as large as those based on *F*, and *R*- factors based on ALL data will be even larger.

### Fractional atomic coordinates and isotropic or equivalent isotropic displacement parameters (Å^2^)

**Table:**

	*x*	*y*	*z*	*U*_iso_*/*U*_eq_	Occ. (<1)
C5	0.28939 (14)	0.14670 (19)	0.53702 (17)	0.0192 (5)	
H5A	0.3403	0.1418	0.5796	0.023*	
H5B	0.2406	0.1491	0.5834	0.023*	
C6	0.28315 (14)	0.0420 (2)	0.46603 (19)	0.0189 (4)	
H6A	0.2881	−0.0283	0.5069	0.023*	
H6B	0.3289	0.0435	0.4152	0.023*	
C7	0.18311 (14)	−0.06318 (18)	0.35971 (17)	0.0172 (4)	
H7A	0.2267	−0.0800	0.3083	0.021*	
H7B	0.1816	−0.1258	0.4101	0.021*	
C8	0.09748 (14)	−0.05164 (18)	0.30595 (17)	0.0171 (4)	
H8A	0.0552	−0.0262	0.3561	0.020*	
H8B	0.0797	−0.1256	0.2778	0.020*	
C9	0.02573 (13)	0.04351 (19)	0.16556 (17)	0.0164 (4)	
H9A	0.0147	−0.0262	0.1257	0.020*	
H9B	−0.0206	0.0546	0.2145	0.020*	
C10	0.03150 (14)	0.14646 (18)	0.09257 (16)	0.0159 (4)	
H10A	−0.0190	0.1508	0.0492	0.019*	
H10B	0.0806	0.1386	0.0472	0.019*	
O1	0.29171 (15)	0.2500	0.47351 (17)	0.0175 (4)	
O2	0.20209 (9)	0.04497 (13)	0.41238 (12)	0.0166 (3)	
O3	0.10476 (9)	0.03158 (13)	0.22212 (12)	0.0156 (3)	
O4	0.03915 (14)	0.2500	0.15568 (16)	0.0148 (4)	
O5	0.11943 (10)	0.14672 (14)	0.73375 (13)	0.0243 (4)	
O6	0.01814 (13)	0.2500	0.83702 (18)	0.0184 (5)	
O7	0.16415 (14)	0.2500	0.88559 (18)	0.0200 (5)	
Cl1	0.10535 (4)	0.2500	0.79722 (6)	0.01469 (17)	
N1	0.18963 (16)	0.2500	0.2839 (2)	0.0142 (5)	
H1A	0.1736	0.1752	0.2975	0.021*	0.50
H1B	0.1462	0.2879	0.2501	0.021*	0.50
H1C	0.2016	0.2870	0.3459	0.021*	0.50
C1	0.4115 (2)	0.2500	0.0932 (2)	0.0179 (6)*	
C2	0.37554 (14)	0.35578 (19)	0.12293 (17)	0.0185 (4)*	
H2A	0.4006	0.4272	0.1007	0.022*	
C3	0.30225 (14)	0.35541 (18)	0.18573 (16)	0.0164 (4)	
H3A	0.2769	0.4268	0.2074	0.020*	
C4	0.26646 (19)	0.2500	0.2164 (2)	0.0139 (5)	
Br1	0.51210 (2)	0.2500	0.00763 (3)	0.02519 (13)	

### Atomic displacement parameters (Å^2^)

**Table:**

	*U*^11^	*U*^22^	*U*^33^	*U*^12^	*U*^13^	*U*^23^
C5	0.0183 (10)	0.0250 (12)	0.0142 (10)	0.0009 (8)	−0.0030 (8)	0.0049 (9)
C6	0.0143 (10)	0.0211 (11)	0.0212 (10)	0.0029 (8)	−0.0023 (8)	0.0037 (9)
C7	0.0202 (10)	0.0131 (9)	0.0184 (10)	0.0006 (8)	0.0010 (8)	0.0021 (8)
C8	0.0199 (10)	0.0141 (10)	0.0173 (10)	−0.0025 (8)	0.0004 (8)	0.0022 (8)
C9	0.0161 (9)	0.0140 (10)	0.0192 (10)	−0.0025 (8)	−0.0022 (8)	−0.0007 (8)
C10	0.0162 (9)	0.0168 (10)	0.0147 (10)	−0.0008 (8)	−0.0021 (8)	−0.0032 (8)
O1	0.0214 (11)	0.0173 (10)	0.0138 (10)	0.000	−0.0018 (8)	0.000
O2	0.0158 (7)	0.0142 (7)	0.0199 (7)	0.0015 (6)	−0.0036 (6)	0.0005 (6)
O3	0.0158 (7)	0.0138 (7)	0.0172 (7)	−0.0020 (5)	−0.0012 (6)	0.0033 (6)
O4	0.0195 (10)	0.0101 (9)	0.0148 (10)	0.000	−0.0032 (8)	0.000
O5	0.0232 (8)	0.0215 (8)	0.0281 (9)	0.0019 (6)	0.0025 (7)	−0.0111 (7)
O6	0.0130 (10)	0.0196 (11)	0.0227 (12)	0.000	0.0015 (8)	0.000
O7	0.0172 (11)	0.0225 (11)	0.0203 (11)	0.000	−0.0038 (9)	0.000
Cl1	0.0135 (3)	0.0137 (3)	0.0168 (3)	0.000	0.0006 (2)	0.000
N1	0.0141 (12)	0.0124 (11)	0.0162 (12)	0.000	−0.0009 (9)	0.000
C3	0.0182 (10)	0.0148 (10)	0.0161 (10)	0.0001 (8)	−0.0017 (8)	−0.0017 (8)
C4	0.0135 (13)	0.0183 (14)	0.0099 (12)	0.000	−0.0025 (10)	0.000
Br1	0.01691 (19)	0.0359 (2)	0.0227 (2)	0.000	0.00469 (11)	0.000

### Geometric parameters (Å, °)

**Table:**

C5—O1	1.431 (2)	C10—H10B	0.9600
C5—C6	1.502 (3)	O1—C5^i^	1.431 (2)
C5—H5A	0.9601	O4—C10^i^	1.435 (2)
C5—H5B	0.9600	O5—Cl1	1.4471 (16)
C6—O2	1.434 (3)	O6—Cl1	1.449 (2)
C6—H6A	0.9601	O7—Cl1	1.444 (2)
C6—H6B	0.9598	Cl1—O5^i^	1.4471 (16)
C7—O2	1.438 (3)	N1—C4	1.470 (4)
C7—C8	1.503 (3)	N1—H1A	0.9100
C7—H7A	0.9599	N1—H1B	0.9100
C7—H7B	0.9600	N1—H1C	0.9100
C8—O3	1.430 (2)	C1—C2	1.388 (3)
C8—H8A	0.9601	C1—C2^i^	1.388 (3)
C8—H8B	0.9601	C1—Br1	1.905 (3)
C9—O3	1.430 (3)	C2—C3	1.391 (3)
C9—C10	1.501 (3)	C2—H2A	0.9500
C9—H9A	0.9600	C3—C4	1.387 (3)
C9—H9B	0.9600	C3—H3A	0.9500
C10—O4	1.435 (2)	C4—C3^i^	1.387 (3)
C10—H10A	0.9600		
			
O1—C5—C6	109.20 (18)	C9—C10—H10A	110.0
O1—C5—H5A	109.9	O4—C10—H10B	110.1
C6—C5—H5A	110.0	C9—C10—H10B	109.9
O1—C5—H5B	109.7	H10A—C10—H10B	108.4
C6—C5—H5B	109.6	C5^i^—O1—C5	111.7 (2)
H5A—C5—H5B	108.3	C6—O2—C7	112.27 (16)
O2—C6—C5	108.66 (17)	C8—O3—C9	111.43 (15)
O2—C6—H6A	110.2	C10^i^—O4—C10	111.7 (2)
C5—C6—H6A	110.2	O7—Cl1—O5^i^	109.40 (9)
O2—C6—H6B	109.8	O7—Cl1—O5	109.40 (9)
C5—C6—H6B	109.7	O5^i^—Cl1—O5	109.88 (15)
H6A—C6—H6B	108.4	O7—Cl1—O6	109.08 (14)
O2—C7—C8	108.39 (17)	O5^i^—Cl1—O6	109.53 (9)
O2—C7—H7A	109.9	O5—Cl1—O6	109.53 (9)
C8—C7—H7A	109.9	C4—N1—H1A	109.5
O2—C7—H7B	110.1	C4—N1—H1B	109.5
C8—C7—H7B	110.1	H1A—N1—H1B	109.5
H7A—C7—H7B	108.4	C4—N1—H1C	109.5
O3—C8—C7	108.84 (16)	H1A—N1—H1C	109.5
O3—C8—H8A	109.9	H1B—N1—H1C	109.5
C7—C8—H8A	109.8	C2—C1—C2^i^	121.9 (3)
O3—C8—H8B	109.8	C2—C1—Br1	119.06 (14)
C7—C8—H8B	110.2	C2^i^—C1—Br1	119.06 (14)
H8A—C8—H8B	108.4	C1—C2—C3	118.9 (2)
O3—C9—C10	109.31 (17)	C1—C2—H2A	120.6
O3—C9—H9A	109.7	C3—C2—H2A	120.6
C10—C9—H9A	110.1	C4—C3—C2	119.5 (2)
O3—C9—H9B	109.8	C4—C3—H3A	120.3
C10—C9—H9B	109.7	C2—C3—H3A	120.3
H9A—C9—H9B	108.3	C3^i^—C4—C3	121.4 (3)
O4—C10—C9	108.34 (17)	C3^i^—C4—N1	119.32 (14)
O4—C10—H10A	110.0	C3—C4—N1	119.32 (14)

Symmetry codes: (i) *x*, −*y*+1/2, *z*.

### Hydrogen-bond geometry (Å, °)

**Table:**

*D*—H···*A*	*D*—H	H···*A*	*D*···*A*	*D*—H···*A*
N1—H1A···O2	0.91	2.13	2.864 (2)	137
N1—H1A···O3	0.91	2.18	2.938 (2)	140
N1—H1B···O4	0.91	2.10	2.850 (3)	139
N1—H1B···O3^i^	0.91	2.20	2.938 (2)	138
N1—H1C···O2^i^	0.91	2.10	2.864 (2)	141
N1—H1C···O1	0.91	2.18	2.875 (3)	133

Symmetry codes: (i) *x*, −*y*+1/2, *z*.
